# Changes in consumer purchasing patterns at New York City chain restaurants following adoption of the sodium warning icon rule, 2015–2017

**DOI:** 10.1371/journal.pone.0274044

**Published:** 2023-04-24

**Authors:** Divya Prasad, John P. Jasek, Amaka V. Anekwe, Christine Dominianni, Tamar Adjoian Mezzacca, Julia S. Sisti, Shannon M. Farley, Kimberly Kessler

**Affiliations:** Bureau of Chronic Disease Prevention, New York City Department of Health and Mental Hygiene, Long Island City, NY, United States of America; University of Rhode Island, UNITED STATES

## Abstract

In 2016, New York City (NYC) began enforcing a sodium warning regulation at chain restaurants, requiring placement of an icon next to any menu item containing ≥2,300 mg sodium. As shifts in consumer purchases are a potential outcome of menu labeling, we investigated whether high-sodium purchases from NYC chains changed following policy implementation. Using receipts for verification, consumer purchases were assessed at 2 full-service (FSR) and 2 quick-service (QSR) chain restaurants in NYC and Yonkers, NY, which did not implement sodium menu labeling, in 2015 and 2017. Primary outcomes included the proportion of respondents purchasing high-sodium item(s) (containing ≥2,300 mg sodium) and mean sodium content of purchases; changes were assessed by difference-in-difference regression models, adjusted for demographic and location co-variates. At both FSR and QSR, there was not a significant change in the proportion of NYC respondents purchasing 1 or more high-sodium items, relative to Yonkers (FSR difference-in-difference: -4.6%, p = 0.364; QSR difference-in-difference: -8.9%, p = 0.196). Among NYC FSR respondents, mean sodium content of purchases significantly declined compared to Yonkers (difference-in-difference: -524 mg, p = 0.012); no changes in mean sodium were observed among QSR participants (difference-in-difference: 258 mg, p = 0.185). Although there was a reduction in mean sodium content of purchases among NYC FSR patrons following sodium warning icon implementation, the mechanism behind the relatively larger NYC decline is unknown.

## Introduction

Sodium in the restaurant environment is problematic, given that restaurant food constitutes more than a quarter of the sodium consumed by US adults [1], the sodium content of restaurant offerings is often high [2, 3], and between 2003–2016, the amount of sodium consumed by adults from full-service restaurant (FSR) meals increased and the amount from fast-food, or quick-service restaurant (QSR) meals, remained high [4]. Average sodium consumption among both US [5] and New York City (NYC) [6] adults far exceeds the 2,300 milligram (mg) Chronic Disease Risk Reduction level established by the National Academies of Sciences [7] and adopted by the Dietary Guidelines for Americans [5], and lowering sodium intake is associated with reductions in hypertension and cardiovascular disease risk [8]. In 2015, approximately 30% of adults in NYC and in the US reported a lifetime diagnosis of hypertension [9, 10], and heart disease accounted for nearly one in five premature deaths in NYC in 2015 [11] and in the US between 2008–2017 [12].

Considering this context, in 2016, NYC began enforcing a sodium warning regulation. A body of research shows that warning labels on foods can increase consumer knowledge and facilitate healthier choices [13] and that improvements in the nutritional composition of chain restaurant menu items may follow menu labeling mandates [14]. This novel policy requires chain restaurants (those with 15 or more locations nationally) to display warning icons next to high-sodium menu items (containing ≥2,300 mg sodium) and post a statement explaining the icon and associated health risks [15] (S1 Fig in S1 File) [16].

Prior to the passage of NYC’s sodium warning regulation, three other US jurisdictions implemented sodium labeling in restaurants: Philadelphia, PA [17], King County, WA [18] and Pierce County, WA [19]. All three efforts involved displaying numerical sodium content, along with other nutrition information such as calories and fat, alongside menu items. Though Philadelphia has since adopted sodium warning icon labeling like NYC’s [20], a consumer purchase evaluation of its previous policy found that the sodium content of purchases at a subset of FSR chains subject to Philadelphia’s then numerical labeling policy was lower than that at control chains outside of Philadelphia [17].

The objective of the current study was to determine whether there were changes in the number of purchases of high-sodium items or changes in the sodium and calorie content of purchases in NYC after the enforcement of the sodium warning regulation. We conducted cross-sectional surveys before and after enforcement began to assess consumer purchases at a subset of FSR and QSR chains in NYC and in Yonkers, NY, a control city.

## Methods

### Study sample

Pre-implementation baseline data were collected at chain restaurants in NYC and Yonkers between October 2015 and January 2016. Yonkers was selected as a control city for its similar demographic profile and existing calorie labeling requirement at chain restaurants, which was also in effect in NYC. Although the sodium warning regulation went into effect in December 2015, enforcement did not begin until June 2016 and none of the chains included in the final analysis implemented the regulation during the baseline data collection period. Post-implementation follow-up data were collected in both cities between April-June 2017.

Rationale and methods behind selection of restaurant chains and locations have been previously described [21]. Briefly, FSR and QSR chains with the greatest number of NYC locations and with at least one location in 3 out of the 5 NYC boroughs and Yonkers were identified as: Applebee’s, IHOP and TGI Friday’s (FSR) and Burger King, McDonald’s, Popeyes and Subway (QSR). From these restaurant chains, at least one FSR and one QSR location in each NYC borough was included. The final analytic sample of restaurants consisted of 2 FSR chains: IHOP and TGI Friday’s, and 2 QSR chains: Popeyes and Subway. Baseline data collection at Applebee’s was terminated early due to this chain’s advance implementation of the sodium warning regulation. Burger King and McDonald’s were excluded from the analytic sample because few or no items met criteria for the sodium warning icon. Following exclusion of these chains, FSR chains still included at least one location per NYC borough, and QSR chains included at least one location in each borough except for Staten Island.

At FSR, surveys were conducted at 9 NYC and 1 Yonkers IHOP locations, and at 4 NYC and 1 Yonkers TGI Friday’s locations. At QSR, surveys were conducted at 3 NYC and 1 Yonkers Popeyes locations, and at 6 NYC (5 at follow-up due to closure of 1 location) and 2 Yonkers Subway locations.

### Measures

#### Survey

Surveys were conducted by trained interviewers between 5–9 pm at FSR locations on weekdays and weekends, and between 12–3 pm at QSR on weekdays. Survey completion targets were 1,000 each at NYC FSR and QSR, and 700 each at Yonkers FSR and QSR at each timepoint. All adult patrons leaving locations were invited to participate in the survey for a $5 incentive. Patrons without an itemized receipt were ineligible. Participants were asked to identify items on their receipt that were purchased for their own consumption, whether and how they modified default menu items (e.g., extra cheese, side salad instead of fries), and whether and how often they refilled a beverage. The survey also collected respondents’ demographic characteristics, including self-reported age group, race/ethnicity, educational level, ZIP code of residence, and interviewer-observed gender. Collecting demographic data by observation is not a best practice at the NYC Department of Health and Mental Hygiene and may have resulted in misclassification since gender cannot be accurately determined by observation.

*Sodium and calories*. Items listed on the receipt that the respondent verified as for their personal consumption were included. Sodium and calorie values for these items were obtained from MenuStat.org (MenuStat), a free, public database that publishes nutrition information from websites of the largest US restaurant chains. This study used nutrition information collected in January 2015 for items purchased at baseline and January 2017 for items purchased at follow-up. Nutrition information could not be obtained for all purchased items, as menu offerings can fluctuate throughout the year or by location, and as MenuStat does not include information for alcoholic beverages. Participants missing nutrition information for their entire purchase were excluded from analysis.

*High-sodium items*. Baseline purchases were coded as “high-sodium items” (S1-S4 Tables in S1 File) if they met criteria for displaying a warning icon–if the item itself had or could be customized to contain 2,300 mg of sodium or more, or if a combination meal was purchased that had the potential to contain at least 2,300 mg of sodium based on the highest-sodium sides that could be included as part of the meal. For example, at Popeyes, all combination meals would exceed 2,300 mg of sodium if the highest sodium side item (mashed potatoes) and the highest sodium beverage (Hawaiian Punch) were selected. Further details on the sodium variations for customizable high-sodium items and combos are provided in the S1-S4 Tables in S1 File footnotes. Photos of menu boards and menu books were taken at a single NYC location for each chain during both data collection periods. If an item purchased at follow-up displayed a sodium warning icon in the restaurant’s follow-up menu photos, it was coded as a high-sodium item. If, at follow-up, a purchased item was not shown on the menu photos, it was coded using the same logic as baseline items.

The NYC Department of Health and Mental Hygiene Institutional Review Board reviewed the study protocol (#15–090) and determined it to be exempt human subjects research on August 31, 2015, under category 2 of the Department of Health and Human Services (HHS) regulations 45 CFR §46.101. While the study was exempt from HHS informed consent requirements, prior to commencing the survey, all potential participants were informed of the general content and time requirement of the survey, that their participation was voluntary and that their answers would be kept private.

### Additional exclusions

**Fig 1:**
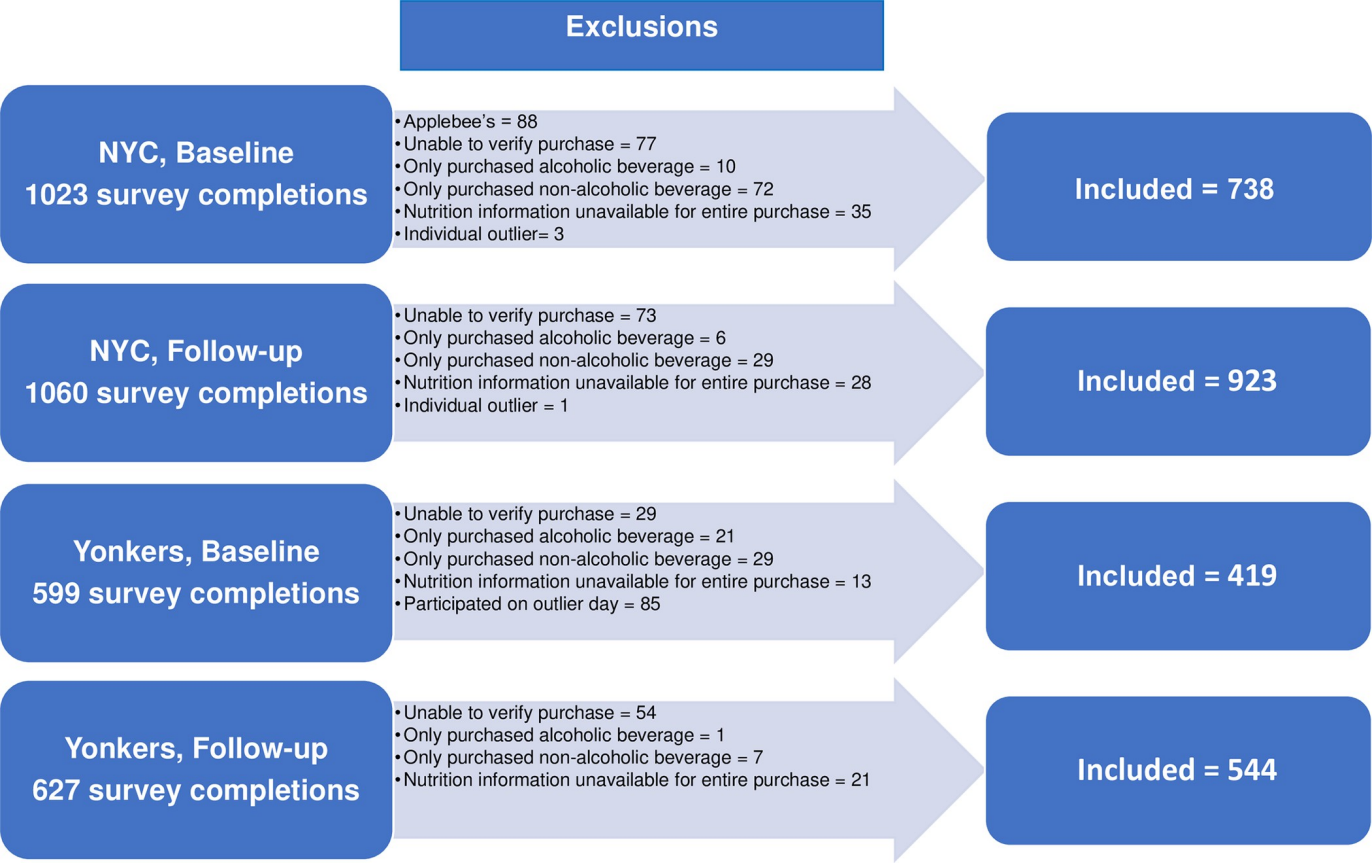
Participant flow chart, full-service restaurants.

**Fig 2:**
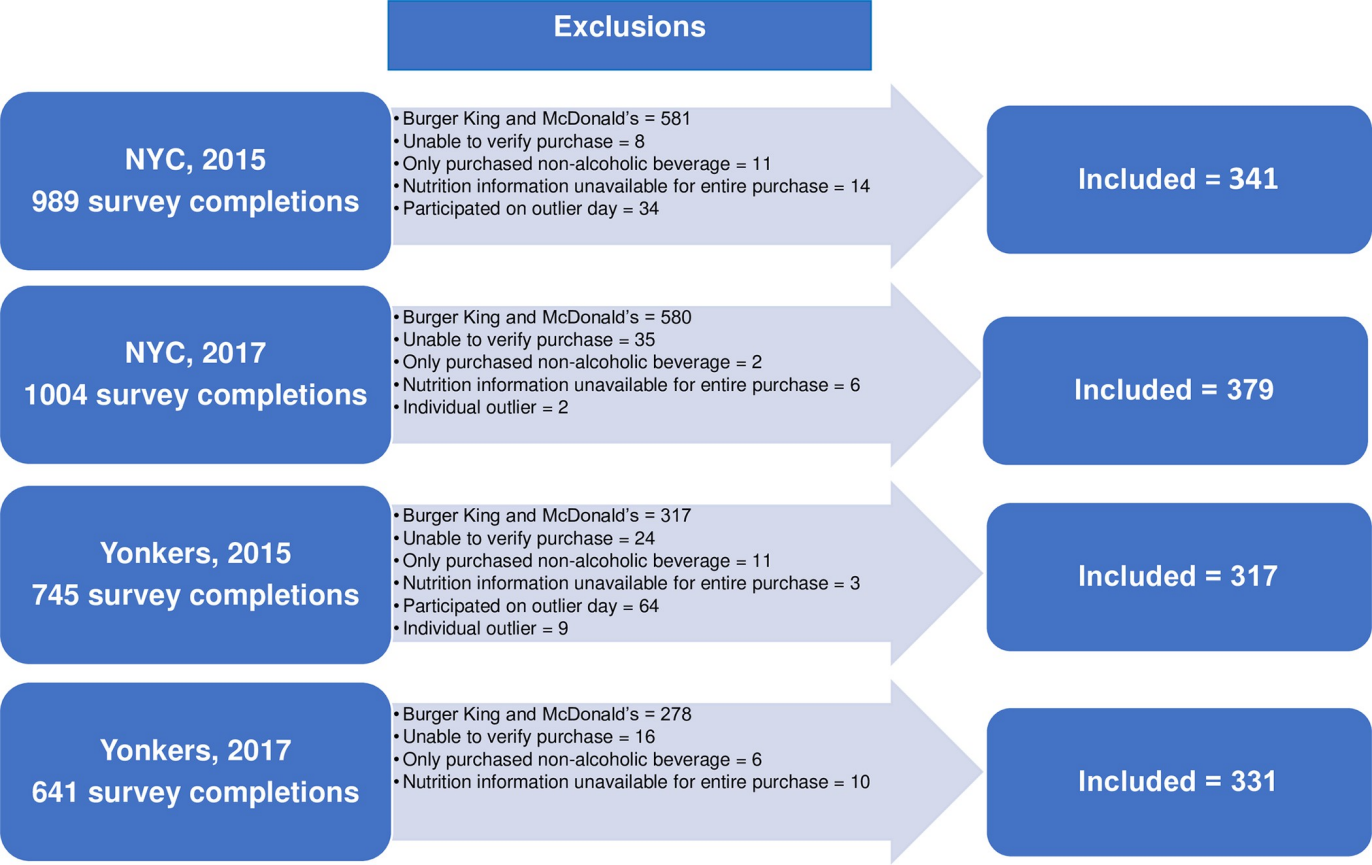
Participant flow chart, quick-service restaurants.

Prior to analysis, participants were excluded if their purchase could not be verified (e.g., items reported as being consumed did not match those listed on the receipt) or if only a beverage was purchased. Extreme outliers, defined as purchases exceeding 12,000 mg of sodium, were excluded. Daily purchases per location were also examined for unusual patterns; “outlier days,” where most purchases were solely for a side item or a beverage, were identified at 1 NYC Subway location, 1 Yonkers Subway location, and 1 Yonkers IHOP location, all at baseline. All purchases made on these dates at these locations were excluded. Figs 1 and 2 show the numbers of FSR and QSR participants excluded for each of these reasons. Age, race/ethnicity, and gender distributions were similar between included and excluded participants. Excluded participants had less educational attainment than included participants.

### Statistical analysis

Sample characteristics were summarized and compared between cities and timepoints via chi-squared tests.

Analyzed outcomes included number of high sodium items purchased, and sodium and calorie content of purchases. Estimates and difference-in-differences between NYC and Yonkers from baseline to follow-up were assessed via mixed-effects regression models. Mean high-sodium items purchased were estimated by Poisson regression. The likelihood of purchasing at least 1 (both FSR and QSR) and at least 2 (FSR only) high-sodium items was assessed using logit regression. At QSR, purchases of 2 or more high-sodium items were extremely rare (among less than 1% of the sample) and therefore were not assessed. Mean sodium and calories purchased were estimated via linear regression models. To reduce skewness and meet model residual normality assumptions, sodium and calorie values were square-root-transformed to assess differences. However, for ease of interpretation, least-squared means estimates from models using non-transformed sodium and calories are reported here. Model estimates for both non-transformed and transformed sodium and calories are shown in S5 Table in S1 File.

All models included fixed effects for restaurant chain, gender, age group, race/ethnicity and education, as these differed between cohorts and each was a significant covariate in modeling for at least one outcome of interest, and random effects for restaurant street address.

Data were analyzed in SAS Enterprise Guide 7.1 (SAS Institute Inc., Cary, NC, USA).

## Results

### Participants

The final sample of respondents consisted of: at NYC FSR, 738 baseline, 923 follow-up; at Yonkers FSR, 419 baseline, 544 follow-up; at NYC QSR, 341 baseline, 379 follow-up; at Yonkers QSR, 317 baseline, 331 follow-up.

Restaurant chain, gender, age, race/ethnicity, location of residence, and educational attainment of participants are summarized for each year and city in Tables 1 (FSR) and 2 (QSR). Sample distribution differed across most of these characteristics significantly between years within each city, and between cities within each year. There were more NYC residents in both the QSR and FSR Yonkers samples at baseline than follow-up; however, when including NYC residency as a co-variate, overall results did not change, nor was NYC residency significant in any model.

**Table 1:** Participant characteristics at baseline and follow-up, full-service restaurants.

	NYC			Yonkers				
	Baseline	Follow-up		Baseline	Follow-up		NYC vs Yonkers	
	n = 738	n = 923		n = 419	n = 544			
	n (%)	n (%)	p value	n (%)	n (%)	p value	p, 2015	p, 2017
**Chain**								
IHOP	631 (85.5)	633 (68.6)	**< .001**	146 (34.8)	495 (91.0)	**< .001**	**< .001**	**< .001**
TGI Friday’s	107 (14.5)	290 (31.4)		273 (65.2)	49 (9.0)			
**Gender**								
Man	385 (52.2)	327 (35.4)	**< .001**	229 (54.7)	205 (37.7)	**< .001**	0.416	0.236
Woman	353 (47.8)	618 (64.6)		190 (45.3)	337 (62.0)			
Other/Unknown	0 (0.0)	0 (0.0)		0 (0.0)	1 (0.2)			
Missing	0 (0.0)	0 (0.0)		0 (0.0)	1 (0.2)			
**Age (years)**						** **		
18–24	181 (24.5)	151 (16.4)	**0.004**	79 (18.9)	36 (6.6)	**< .001**	0.133	**< .001**
25–34	194 (26.3)	265 (28.7)		117 (27.9)	96 (17.6)			
35–44	199 (27.0)	276 (29.9)		115 (27.4)	238 (43.8)			
45–64	142 (19.2)	197 (21.3)		87 (20.8)	135 (24.8)			
65+	21 (2.9)	32 (3.5)		21 (5.0)	39 (7.2)			
Refused	1 (0.1)	2 (0.2)		0 (0.0)	0 (0.0)			
**Race/Ethnicity**						** **		** **
Asian/Pacific Islander, non-Latino	46 (6.2)	18 (2.0)	**< .001**	12 (2.9)	6 (1.1)	0.270	**< .001**	**< .001**
Black, non-Latino	241 (32.7)	359 (38.9)		169 (40.3)	224 (41.2)			
Latino	264 (35.8)	314 (34.0)		100 (23.9)	130 (23.9)			
White, non-Latino	148 (20.1)	205 (22.2)		123 (29.4)	170 (31.3)			
Other, non-Latino	36 (4.9)	22 (2.4)		14 (3.3)	11 (2.0)			
Don’t Know/Refused	3 (0.4)	5 (0.5)		1 (0.2)	3 (0.6)			
**Education**						** **		** **
Less than HS	38 (5.2)	19 (2.1)	**< .001**	17 (4.1)	3 (0.6)	**< .001**	0.238	**< .001**
High School Grad	223 (30.2)	270 (29.3)		147 (35.1)	124 (22.8)			
Some College	268 (36.3)	280 (30.3)		153 (36.5)	234 (43.0)			
College Grad	209 (28.3)	353 (38.2)		102 (24.3)	182 (33.5)			
Refused	0 (0.0)	1 (0.1)		0 (0.0)	1 (0.2)			
**Residence**								
NYC	627 (85.0)	817 (88.5)	**< .001**	3 (0.7)	74 (13.6)	**< .001**	**< .001**	**< .001**
Yonkers	30 (4.1)	9 (1.0)		382 (91.2)	410 (75.4)			
Other	81 (11.0)	94 (10.2)		34 (8.1)	60 (11.0)			

Baseline data were collected between October 2015-January 2016. Follow-up data were collected between April-June 2017.

**Bolded** p values indicate statistically significant differences (p < 0.05).

**Table 2:** Participant characteristics at baseline and follow-up, quick-service restaurants.

	NYC			Yonkers				
	Baseline	Follow-up		Baseline	Follow-up		NYC vs Yonkers	
	n = 341	n = 379		n = 317	n = 331			
	n (%)	n (%)	p value	n (%)	n (%)	p value	p, 2015	p, 2017
**Chain**								
Popeyes	107 (31.4)	146 (38.5)	**0.045**	307 (96.8)	170 (51.4)	**< .001**	**< .001**	**< .001**
Subway	234 (68.6)	233 (61.5)		10 (3.2)	161 (48.6)			
**Gender**								
Man	189 (55.4)	178 (47.0)	**0.040**	145 (45.7)	150 (45.3)	0.914	**0.026**	0.660
Woman	151 (44.2)	201 (53.0)		172 (54.3)	181 (54.7)			
Other/Unknown	0 (0.0)	0 (0.0)		0 (0.0)	0 (0.0)			
Missing	1 (0.3)	0 (0.0)		0 (0.0)	0 (0.0)			
**Age (years)**			** **					
18–24	72 (21.1)	59 (15.6)	**0.004**	57 (18.0)	27 (8.2)	**< .001**	0.338	**0.010**
25–34	85 (24.9)	100 (26.4)		71 (22.4)	86 (26.0)			
35–44	66 (19.4)	118 (31.1)		83 (26.2)	133 (40.2)			
45–64	104 (30.5)	89 (23.5)		93 (29.3)	78 (23.6)			
65+	13 (3.8)	13 (3.4)		13 (4.1)	7 (2.1)			
Refused	1 (0.3)	0 (0.0)		0 (0.0)	0 (0.0)			
**Race/Ethnicity**			** **			** **		** **
Asian/Pacific Islander, non-Latino	12 (3.5)	11 (2.9)	**< .001**	1 (0.3)	2 (0.6)	**0.048**	**< .001**	0.158
Black, non-Latino	137 (40.2)	193 (50.9)		151 (47.6)	190 (57.4)			
Latino	104 (30.5)	125 (33.0)		121 (38.2)	104 (31.4)			
White, non-Latino	64 (18.8)	45 (11.9)		35 (11.0)	30 (9.1)			
Other, non-Latino	22 (6.5)	3 (0.8)		9 (2.8)	3 (0.9)			
Don’t Know/Refused	2 (0.6)	2 (0.5)		0 (0.0)	2 (0.6)			
**Education**			** **			** **		** **
Less than HS	25 (7.3)	21 (5.5)	0.133	23 (7.3)	8 (2.4)	**< .001**	**< .001**	**< .001**
High School Grad	115 (33.7)	134 (35.4)		120 (37.9)	98 (29.6)			
Some College	82 (24.1)	118 (31.1)		112 (35.3)	156 (47.1)			
College Grad	118 (34.6)	105 (27.7)		62 (19.6)	69 (20.9)			
Refused	1 (0.3)	1 (0.3)		0 (0.0)	0 (0.0)			
**Residence**								
NYC	298 (87.4)	358 (94.5)	**< .001**	26 (8.2)	84 (25.4)	**< .001**	**< .001**	**< .001**
Yonkers	1 (0.3)	6 (1.6)		267 (84.2)	207 (62.5)			
Other	41 (12.0)	15 (4.0)		24 (7.6)	40 (12.1)			

Baseline data were collected between October 2015-January 2016. Follow-up data were collected between April-June 2017.

**Bolded** p values indicate statistically significant differences (p < 0.05).

### High-sodium item purchases (Table 3)

**Table 3:** High-sodium dinnertime purchases at full-service and lunchtime purchases at quick-service restaurant chains, pre- (baseline) and post- (follow-up) implementation of the sodium warning icon in NYC.

	NYC vs Yonkers	NYC (intervention)						Yonkers (control)					
	Difference In Difference*	Baseline			Follow-up			Baseline			Follow-up		
**Full-Service Restaurants**	** **	n = 734			n = 915			n = 418			n = 538		
# High sodium items purchased per participant, mean (95% CI)	-0.13	0.83	(0.74,	0.93)	**0.65^a^**	(0.58,	0.73)	0.67	(0.59,	0.76)	0.62	(0.53,	0.71)
Participants purchasing at least 1 high-sodium item, % (95% CI)	-4.6	67.4	(61.1,	73.1)	65.0	(58.6,	70.8)	59.3	(51.5,	66.7)	61.5	(53.8,	68.7)
Participants purchasing at least 2 high-sodium items, % (95% CI)	-7.5	15.0	(9.9,	22.0)	**3.1^a^**	(1.7,	5.4)	6.1	(3.6,	10.3)	**1.7^b^**	(0.6,	4.4)
**Quick-Service Restaurants**	** **	n = 338			n = 376			n = 317			n = 329		
# High sodium items purchased per participant, mean (95% CI)	-0.03	0.25	(0.19,	0.34)	0.27	(0.20,	0.36)	0.25	(0.19,	0.34)	0.30	(0.22,	0.41)
Participants purchasing at least 1 high-sodium item, % (95% CI)	-8.9	26.8	(18.2,	37.7)	30.3	(20.8,	41.9)	24.6	(16.3,	35.5)	**37.0^b^**	(25.7,	50.0)

Baseline data were collected between October 2015-January 2016. Follow-up data were collected between April-June 2017.

Models were adjusted for restaurant chain, gender, age group, education, and race/ethnicity, with random effects for restaurant location (street address).

Sample sizes for participants included in the models are shown. Due to missingness in fixed effects variables, 19 FSR and 8 QSR participants in the final analytic sample were not included in modeled estimates.

* all p values for difference-in-differences were greater than 0.05.

**Bolded** estimates indicate statistically significant differences between baseline and follow-up.

^a^ 2017 vs 2015 p < 0.001.

^b^ 2017 vs 2015 p < 0.05.

Among NYC FSR respondents, there was a significant decline from baseline to follow-up in the mean number of high-sodium items purchased (0.83 vs 0.65 items/participant, p<0.001) and the proportion of respondents purchasing at least 2 high-sodium items (15% vs 3%, p<0.001). The proportion of respondents purchasing at least 1 high-sodium item did not differ between baseline and follow-up (67% vs 65%, p = 0.389).

Among Yonkers FSR respondents, there was a significant decline from baseline to follow-up in the proportion of respondents purchasing at least 2 high-sodium items (6% vs 2%, p = 0.014). The mean number of high-sodium items purchased by Yonkers FSR respondents did not differ between baseline and follow-up (0.67 vs 0.62 items/participant, p = 0.309), nor did the proportion of respondents purchasing at least 1 high-sodium item (59% vs 62%, p = 0.598).

At FSR, there was no significant change from baseline to follow-up in NYC relative to Yonkers (i.e., difference-in-difference) in the mean number of high-sodium items purchased (p = 0.120) or in the proportion of respondents purchasing at least 1 (p = 0.364) or at least 2 (p = 0.509) high-sodium items.

Among NYC QSR respondents, there was no significant difference between baseline and follow-up in the mean number of high-sodium items purchased (0.25 vs 0.27 items/participant, p = 0.603), or in the proportion of participants purchasing at least one of these items (27% vs 30%, p = 0.422).

Among Yonkers QSR participants, there was a significant increase from baseline to follow-up (25% vs 37%, p = 0.027) in the proportion of participants purchasing at least one high-sodium item. The mean number of high-sodium items did not significantly differ between baseline and follow-up (0.25 vs 0.30 items/participant, p = 0.112).

At QSR, there was no difference-in-difference between NYC and Yonkers in the mean number of high-sodium items purchased (p = 0.463) or in the proportion of respondents purchasing at least 1 high-sodium item (p = 0.196). Results for high-sodium purchases at individual chains are shown in S6 Table in S1 File.

#### Sodium and calories

**Fig 3:**
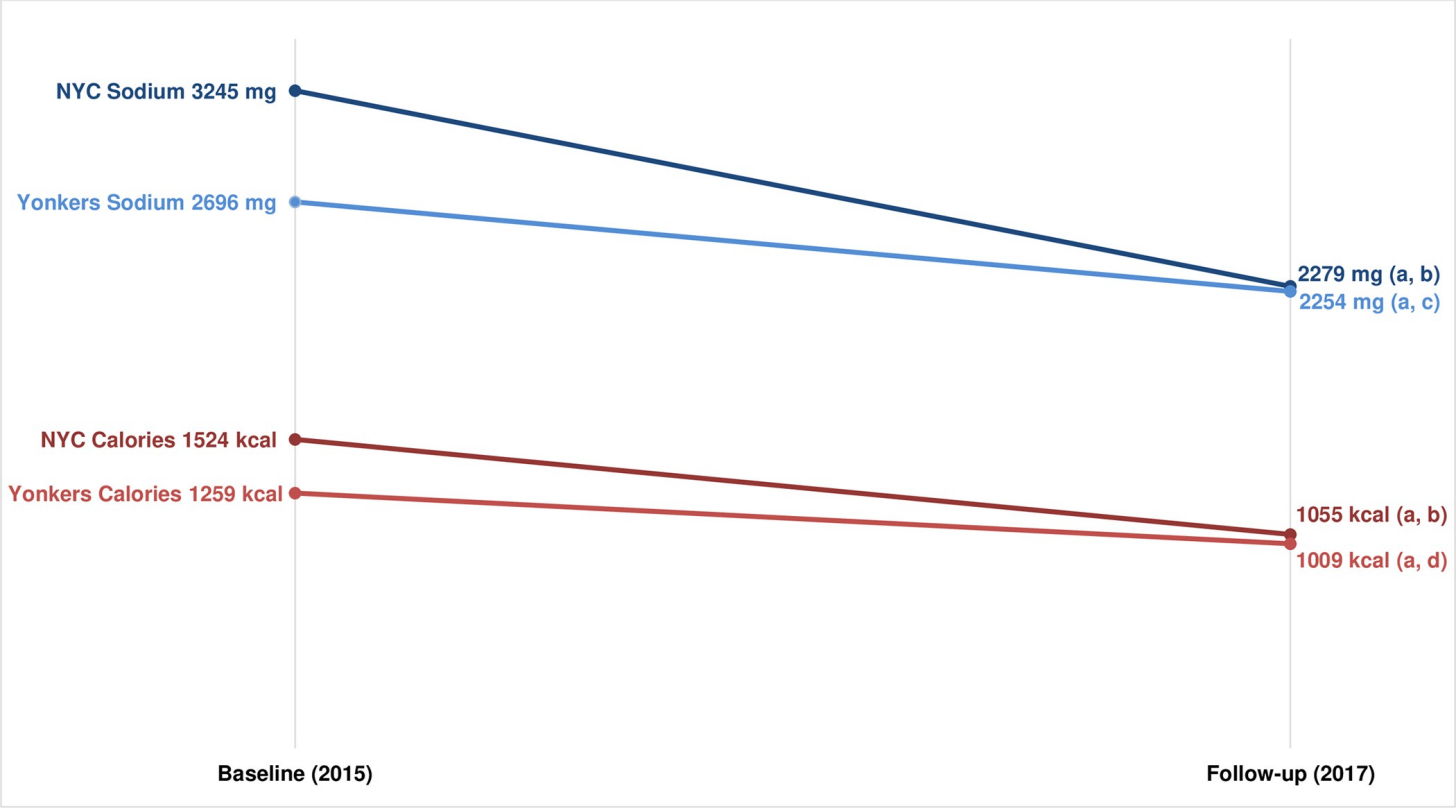
Mean sodium and calorie content* of dinnertime purchases at FSR chains, pre- (Baseline) and post- (Follow-up) implementation of the sodium warning icon in NYC. Baseline data were collected between October 2015-January 2016. Follow-up data were collected between April-June 2017. *Sodium and calorie values were square-root-transformed during analysis to meet model normality assumptions and to estimate differences (p values), but least-squared means estimates from models using non-transformed variables are presented for ease of interpretation. Full model estimates are shown in S5 Table in S1 File. a) NYC vs Yonkers Difference-in-Difference p value < 0.05; b) 2015–16 vs 2017 p value < 0.001; c) 2015–16 vs 2017 p value < 0.05; d) 2015–16 vs 2017 p value <0.01.

Mean sodium content of purchases declined significantly from baseline to follow-up at both NYC FSR (3,245 mg vs 2,279 mg, p <0.001) and Yonkers FSR (2,696 mg vs 2,254 mg, p = 0.016). The decline in NYC was significantly greater than that in Yonkers (p = 0.012). Similar patterns were present for the mean calorie content of purchases, with declines at both NYC (baseline: 1,524 kcal; follow-up: 1,055 kcal; p <0.001) and Yonkers (baseline: 1,259 kcal; follow-up: 1,009 kcal; p = 0.004), and a significantly greater decline in NYC than in Yonkers (p = 0.02) (Fig 3).

**Fig 4:**
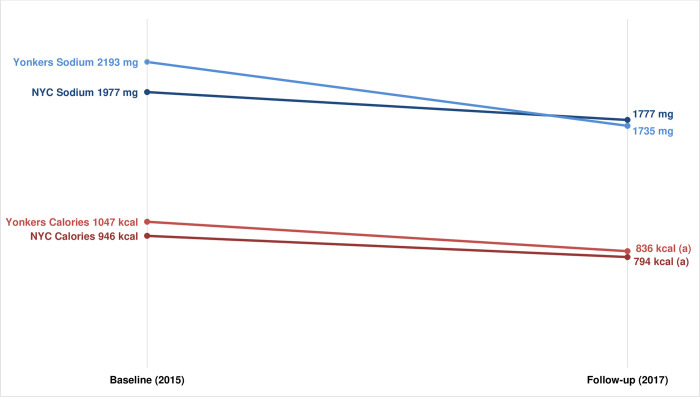
Mean sodium and calorie content* of lunchtime purchases at QSR chains, pre- (Baseline) and post-(Follow-up) implementation of the sodium warning icon in NYC. *Sodium and calorie values were square-root-transformed during analysis to meet model normality assumptions and to estimate differences (p values), but least-squared means estimates from models using non-transformed variables are presented for ease of interpretation. Full model estimates are shown in S5 Table in S1 File. a) 2015–16 vs 2017 p value < 0.05.

At QSR, mean sodium content of purchases did not significantly differ from baseline to follow-up in NYC (1,977 mg vs 1,777 mg, p = 0.162) or in Yonkers (2,193 mg vs 1,735 mg, p = 0.064), and there was no difference-in-difference between the two cities (p = 0.570). Mean calories purchased declined from baseline to follow-up in both NYC (946 kcal vs 794 kcal, p = 0.013) and in Yonkers (1,047 kcal vs 836 kcal, p = 0.039), with no difference-in-difference between the two cities (p = 0.768) (Fig 4). Sodium results for individual chains are shown in S7 Table in S1 File.

## Discussion

Here, we find mixed evidence of changes in purchasing patterns at NYC FSR following implementation of the sodium warning icon. Although decreases in purchases of high-sodium items among NYC FSR respondents were not significant relative to changes in purchases made by Yonkers respondents, both the mean sodium and calorie content of purchases made at NYC FSR declined significantly compared to Yonkers. This change may have been driven by the substantial reduction in the mean number of high-sodium items purchased by NYC FSR patrons. However, at baseline, FSR purchases of high-sodium items and mean sodium content were much higher in NYC than in Yonkers for unknown reasons. These analyses accounted for potentially confounding covariates such as demographic characteristics, which significantly differed between cities/years, and excluded extreme outliers. While numeric calorie amounts were listed next to all menu items in both cities and at both timepoints, media attention prior to the implementation of federal calorie labeling regulations that expanded the reach of calorie labels to chain retail establishments in 2018 may have heightened awareness of calorie information on chain restaurant menus, which could have led to lower calorie and lower sodium purchases captured at follow-up. Additionally, other factors such as location-specific promotions may have contributed to the baseline purchasing differences between NYC and Yonkers.

At QSR, there were no observed changes in high-sodium item purchases or in the sodium or calorie content of purchases in NYC compared to Yonkers. If the sodium warning icon did play a role in the reductions observed at NYC FSR, it is possible that a parallel impact was not seen in the QSR environment because of the differences in ordering method; at FSR, patrons presumably spend a longer time reviewing the menu book than QSR patrons may spend looking at the menu board. Further, QSR purchases were made during lunchtime hours (12–3 pm), while FSR purchases were made during dinnertime hours (5–9 pm). Additionally, the sodium warning icon may have less utility at QSR in general due to fewer items meeting the 2,300 mg requirement, as Sisti et al. found in the accompanying menu evaluation [22], and as was found in the present study, where we excluded two QSR chains because of low or no warning icon presence. However, if sodium warning icons are ubiquitous on a restaurant’s menu, as it the case in some FSR chain restaurants, then they may also be less impactful, as consumers will have few lower-sodium alternatives.

While this study is, to our knowledge, the only real-world evaluation of consumer purchases following implementation of the NYC sodium warning regulation, two studies of hypothetical purchasing responses to sodium warning icons among online panels have been conducted. Byrd et al. tested whether, when presented with sodium labeling, hypothetical meal choices differed across taste preferences, with null findings for NYC’s sodium warning icon and mixed results for numeric sodium labeling [23]. Musicus et al. evaluated the effectiveness of various sodium warning icon designs, with positive findings for icons that included “Sodium Warning” text [24].

During data collection, NYC was the only jurisdiction in the United States with sodium warning labeling in the restaurant environment. Since then, Philadelphia implemented a similar sodium warning policy (their design informed by Musicus et al.’s research) in chain restaurants in 2019 [20], although no subsequent evaluations have been published.

### Limitations

The present study has several limitations. Findings cannot be generalized to the NYC population or to NYC FSR and QSR chains overall, as the sample was limited to patrons at 2 FSR and 2 QSR chains. There is a potential for selection bias, as convenience sampling was utilized, and only those with an itemized receipt were eligible. Participation rates were not documented. Although participants indicated which items were for their personal consumption, it is unknown how much they consumed. We also did not collect information on the number of people that participants shared food with. While chain restaurants with consistent menus nationwide were chosen, it is possible that promotions and pricing differed across locations. Baseline and follow-up data were collected during different seasons, with baseline data collection occurring during fall and early winter, and follow-up during spring; although conducting a difference-in-differences analysis with a control city helps mitigate this limitation, changes from baseline to follow-up may have been influenced by the seasonal change. The FSR baseline sample size was smaller than that at follow-up due to the exclusion of Applebee’s, exclusion of more respondents for only purchasing beverages, and exclusion of respondents who participated on an outlier day, and the QSR sample was substantially reduced at both timepoints due to exclusion of Burger King and McDonald’s, thus reducing the power of the study to detect smaller changes. Finally, the study was not adequately sampled to examine results by chain, or whether purchases differed by race, ethnicity, and other socio-demographic factors.

## Conclusions

While efforts like nutrient warnings aim to foster transparency about the food supply and may contribute to health-promoting behaviors [25], commercial factors such as excessive marketing, targeted marketing, and unhealthy food offerings can strongly influence health [26]. The accompanying menu evaluation by Sisti et al. found no reduction in high-sodium offerings at chain restaurants approximately one year following implementation of the sodium warning icon [22]; it may take additional time and action to reduce sodium in restaurant foods. Recently, the US Food and Drug Administration released voluntary sodium reduction goals for the food industry [27]. This, along with restaurant sodium labeling policies from additional jurisdictions, may prompt reformulation and lower- sodium offerings in the restaurant environment, which may have a larger effect on consumer purchasing. Further research on longer-term changes following such policies is warranted. Additionally, as new menu labeling policies are debated and passed (most recently, NYC’s Sweet Truth Act [28]), ongoing evaluation of label designs should be conducted to maximize the likelihood of their usefulness to consumers.

## Supporting information

S1 File This file contains S1 Fig and S1-S7 Tables. (DOCX)
