# Supplementary material for: Changes in consumer purchasing patterns at New York City chain restaurants following adoption of the sodium warning icon rule, 2015–2017
Source: PLoS One. 2023 Apr 24;18(4):e0274044. doi: 10.1371/journal.pone.0274044 (PMC10124888; doi:10.1371/journal.pone.0274044)
Supplement: S1 File — (DOCX) [file pone.0274044.s001.docx]

**Supporting Information:** **Changes in consumer purchasing patterns at New York City chain restaurants following adoption of the sodium warning icon rule, 2015-2017**

S1 Fig: NYC chain restaurant sodium warning icon 2

S1 Table: High-sodium items, IHOP, 2015 and 20173

S2 Table: High-sodium items, TGI Friday’s, 2015 and 20175

S3 Table: High-sodium items, Popeyes, 2015 and 20177

S4 Table: High-sodium items, IHOP, 2015 and 20178

S5 Table: Square root-transformed sodium and calorie content of purchases at full-service and quick-service restaurant chains, pre- (baseline) and post- (follow-up) implementation of the sodium warning icon in NYC9

S6 Table: High-sodium dinnertime purchases at IHOP and TGI Friday's and lunchtime purchases at Popeyes, pre- (baseline) and post- (follow-up) implementation of the sodium warning icon in NYC …………………...10

S7 Table: Sodium content of dinnertime purchases at IHOP and TGI Friday's and lunchtime purchases at Popeyes, pre- (baseline) and post- (follow-up) implementation of the sodium warning icon in NYC………….11

**S1 Fig: NYC chain restaurant sodium warning icon (a), displayed next to all items containing 2,300 mg of sodium or more, and (b) sodium warning statement, posted in each restaurant at the point of order.**

1. Sodium warning icon placed next to all items containing 2,300 mg of sodium or more


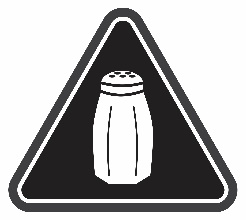


1. Sodium warning statement, posted in each restaurant at the point of order

**
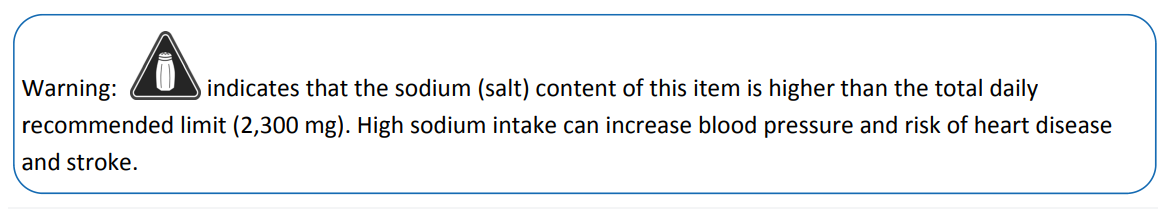
**

**S1 Table: High-sodium items*, IHOP, 2015 and 2017**

| **Included item*** | **Sodium content, mg**** | **Notes** |
| --- | --- | --- |
| Appetizer Sampler | 3,520 |  |
| Bacon & Eggs Combo ^a^ | 2,215 - 2,420 |  |
| Bacon Cheeseburger ^b^ | 1,760 - 2,500 |  |
| Bacon Crusted Chicken Breast with Potato Hash ^c^ | 2,175 - 3,260 | only offered in 2017 |
| Bacon Lettuce Tomato Avocado (BLTA) | not available | only offered in 2017 |
| Bacon Temptation Omelette ^d^ | 2,310 - 3,840 |  |
| Big 3-Egg Breakfast ^e^ | 2,040 - 3,780 | only offered in 2017 |
| Big Steak Omelette ^d^ | 2,030 - 3,560 |  |
| Blue Cheese & Bacon Burger ^b^ | 1,670 - 2,410 |  |
| Breakfast Sampler ^f^ | 2,785 - 2,920 |  |
| Californian Scramble ^g^ | 1,740 - 2,840 |  |
| Cheeseburger Omelette ^d^ | 3,170 - 4,700 | only offered in 2017 |
| Cheeseburger Sliders ^b^ | 2,380 - 3,120 | only offered in 2017 |
| Chicken & Spinach Salad ^h^ | 2,170 - 2,830 |  |
| Chicken & Three-Cheese Quesadilla | 2,400 |  |
| Chicken Clubhouse Super Stacker ^b^ | 3,330 - 4,070 |  |
| Chicken Cobb Salad ^h^ | 1,940 - 2,370 |  |
| Chicken Cordon Bleu ^c^ | 2,765 - 3,950 |  |
| Chicken Fajita Omelette ^d^ | 2,160 - 3,690 |  |
| Chicken Verde & Sweet Corn Tamale Cakes | not available | only offered in 2017 |
| Chorizo Fiesta Omelette ^d^ | 3,220 - 5,050 | only offered in 2015 |
| Classic Breakfast Crepes | 2,400 |  |
| Colorado Omelette ^d^ | 2,750 - 4,280 |  |
| Corned Beef Hash & Cheese Omelette ^d^ | 2,000 - 3,530 |  |
| Corned Beef Hash & Eggs ^i^ | 2,670 - 2,870 |  |
| Country Fried Steak & Eggs ^f^ | 3,695 - 3,830 |  |
| Country Fried Steak ^c^ | 2,855 - 4,380 |  |
| Country Omelette ^d^ | 2,000 - 3,530 |  |
| Create Your Perfect Omelette ^d^ | 640 - 2,935 |  |
| Crispy Chicken Strips & Fries | 2,360 |  |
| Denver Omelette Burger ^b^ | 2,210 - 2,950 | only offered in 2017 |
| Double BLT ^b^ | 2,080 - 2,820 |  |
| Fried Chicken Dinner ^c^ | 3,955 - 5,240 |  |
| Garden Omelette ^d^ | 1,050 - 2,580 |  |
| Garlic Butter Chicken & Potato Pancakes | not available | Only offered in 2017 |
| Grilled Chicken, Ranch & Avocado Club ^b^ | 1,900 - 2,640 | only offered in 2017 |
| Ham & Egg Melt ^b^ | 2,900 - 3,640 |  |
| Ham & Eggs Combo  ^a^ | 2,765 - 2,970 |  |
| Hearty Ham & Cheese Omelette ^d^ | 2,460 - 3,790 |  |
| Hot & Spicy Wings | 4,610 | only offered in 2017 |
| Loaded Potato Pancakes | not available | only offered in 2017 |
| Mega Monster Cheeseburger ^b^ | 1,680 - 2,420 |  |
| Monster Mozza Sticks | 2,680 |  |
| Patty Melt ^b^ | 1,920 - 2,660 |  |
| Philly Cheese Steak Stacker ^b^ | 2,590 - 3,330 |  |
| Roasted Turkey & Fixings ^c^ | 2,585 - 3,770 |  |
| Roasted Turkey Sandwich ^b^ | 1,910 - 2,650 | only offered in 2017 |
| Rustic Smokehouse Hash | not available | only offered in 2017 |
| Sausage & Eggs Combo ^a^ | 2,105 - 2,310 |  |
| Sirloin Steak Tips ^c^ | 2,475 - 3,760 |  |
| Sirloin Tips & Eggs ^f^ | 2,895 - 3,030 |  |
| Smokehouse Combo ^f^ | 2,765 - 2,900 |  |
| Southwestern Club Sandwich ^b^ | 2,440 - 2,930 | only offered in 2015 |
| Spicy Chicken Ranch Sandwich ^b^ | 2,410 - 3,150 |  |
| Spinach & Mushroom Omelette ^d^ | 1,630 - 3,160 |  |
| T-Bone Steak & Eggs ^i^ | 2,360 - 2,560 |  |
| Tilapia Florentine ^c^ | 2,405 - 3,590 |  |
| Turkey, Bacon & Avocado Wrap ^b^ | 2,100 - 2,840 | only offered in 2017 |
| Tuscan Scramble ^g^ | 1.400 - 2,500 |  |
| Ultimate Bacon & Sausage Combo ^a^ | 2,485 - 2,690 |  |

*Included items that either: 1) displayed a warning icon in 2017 at the time of data collection, or 2) were not on the menu in 2017, but were ordered in 2015 and contained >= 2,300 mg sodium.

**For items not on the menu in 2017, the 2015 sodium content is listed. Otherwise, the listed sodium content values are from 2017.

The following footnotes apply to items which list a range of sodium:

^a^ Pancake combo with choice of 2 eggs and 2 pancakes: Lowest sodium egg option was 2 Hard or Soft Boiled Eggs (125 mg); highest sodium egg option was 2 Poached Eggs (260 mg). Lowest sodium pancake option was 2 Buttermilk Pancakes w/Butter (950 mg); highest sodium pancake option was 2 Red Velvet Cheesecake Pancakes (1,020 mg).

^b^ Burgers & Sandwiches: Lowest sodium side was Fruit (0 mg); highest sodium side was French Fries (2017: 740 mg; 2015: 490 mg).

^c^ Entrée: Lowest sodium side was Baked Potato (15 mg); highest sodium side was Rustic Cheesy Tomato Soup (1,200 mg).

^d^ Omelette: Lowest sodium side was Fruit (0 mg); highest sodium side was 3 New York Cheesecake Pancakes (2017: 1,530 mg; 2015: 1,830 mg).

^e^ Egg Combo with choice of 3 eggs, choice of meat, and optional hash browns. Lowest sodium egg option was 3 Hard or Soft Boiled Eggs (190 mg); highest sodium egg option was 3 Poached Eggs (390 mg). Lowest sodium meat option was 2 Pork Chops (4oz) (460 mg); highest sodium meat option was 4 Spam® (1570 mg). Hash Browns (430 mg) included in high end of range and not included in low end of range.

^f^ Egg Combo with 2 eggs: Lowest sodium egg option was 2 Hard or Soft Boiled Eggs (125 mg); highest sodium egg option was 2 Poached Eggs (260 mg).

^g^ Made to Crack Egg Specialty with Choice of Potato and Side: Lowest sodium potato option was Hash Browns (430 mg); highest sodium potato option was Red Potato Hash (840 mg). Lowest sodium side was White Toast w/Butter & Jam or Jelly (260 mg); highest sodium side was 2 buttermilk pancakes (950 mg).

^h^ Salads: Lower sodium option included Grilled Chicken; higher sodium option included Crispy Chicken.

^i^ Egg Combo with 3 eggs: Lowest sodium egg option was 3 Hard or Soft Boiled Eggs (190 mg); highest sodium egg option was 3 Poached Eggs (390 mg).

**S2 Table: High-sodium items*, TGI Friday's, 2015 and 2017**

| **Included Item*** | **Sodium content, mg**** | **Notes** |
| --- | --- | --- |
| 10 oz New York Strip ^a^ | 1,610 - 2,740 |  |
| All-American Stacked Burger | 4,060 |  |
| Baby Back Ribs | 2,420 (half rack)/ 3,010 (full rack) |  |
| Boneless Wings ^b^ | 2,340 - 3,960 |  |
| Bourbon Barrel Chicken ^a^ | 1,320 - 2,450 |  |
| Bourbon Barrel Mahi Mahi ^a^ | 1,060 - 2,190 | Only offered in 2017 |
| Cajun Shrimp & Chicken Pasta | 2,410 |  |
| California Chicken Club ^c^ | 2,385 - 3,160 |  |
| Classic Cheeseburger | 3,510 |  |
| Crispy Chicken Fingers | 2,760 |  |
| Flat Iron & Langostino Lobster Topping ^a^ | 1,830 -2,960 |  |
| Flat Iron ^a^ | 1,290 - 2,420 |  |
| French Dip ^c^ | 1,785 - 2,560 |  |
| French Onion Soup | 2,550 |  |
| Friday's Pick 2 Combo ^d^ | 870 - 4,380 |  |
| Friday's Shrimp | 3,320 |  |
| Grilled Norweigian Salmon with Langostino Lobster ^a^ | 1,680 - 2,810 |  |
| Jack Daniel's Burger | 4,070 |  |
| Jack Daniel's Chicken & Ribs | not available |  |
| Jack Daniel's Chicken & Shrimp ^a^ | 2,340 - 3,470 |  |
| Jack Daniel's Chicken ^a^ | 2,090 - 3,220 |  |
| Jack Daniel's Chicken Sandwich ^c^ | 3,205 - 3,980 |  |
| Jack Daniel's Flat Iron & Ribs ^a^ | 2,500 - 3,630 |  |
| Jack Daniel's Flat Iron ^a^ | 1,740 - 2,870 |  |
| Jack Daniel's New York Strip & Shrimp ^a^ | 2,650 - 3,780 |  |
| Jack Daniel's New York Strip ^a^ | 1,730 - 2,860 |  |
| Jack Daniel's Rib-eye & Shrimp ^a^ | 2,690 - 3,820 |  |
| Jack Daniel's Rib-eye ^a^ | 1,770 - 2,900 |  |
| Jack Daniel's Ribs | 3,080 |  |
| Jack Daniel's Ribs & Shrimp | 4,000 |  |
| Jack Daniel's Sampler | 4,390 |  |
| Jack Sliders ^e^ | 2,360 - 4,280 |  |
| Kid's Chicken Fingers ^f^ | 1,165 - 2,305 |  |
| Kid's Mac & Cheese ^f^ | 1,225 - 2,365 |  |
| Kid's Sliders ^f^ | 1,295- 2,435 |  |
| Mozzarella Sticks ^ǂ^ | 1,180 |  |
| New York Cheddar & Bacon Burger | 4,280 |  |
| Rib-eye & Baby Back Ribs ^a^ | 2,490 - 3,620 |  |
| Rib-eye ^a^ | 1,320 - 2,450 |  |
| Sedona Black Bean Burger | 3,190 |  |
| Sesame Jack Chicken Strips | 2,700 |  |
| Sicilian Burger | 4,730 | Only offered in 2015 |
| Smoke Stacked Burger | 4,180 |  |
| Spicy Chicken Sliders ^e^ | 1,800 - 3,600 | Only offered in 2017 |
| The Friday's Combo | not available | Only offered in 2017 |
| Traditional Wings ^b^ | 1,620 - 3,560 |  |
| Triple Stack Rueben ^c^ | 3,535 - 4,170 | Only offered in 2015 |
| Turkey Burger | 2,760 |  |
| Warm Pretzels with Craft Beer-Cheese Dipping Sauce | 3,170 |  |
| Your Call Burger ^g^ | 3,280 - 4,465 |  |

*included items that either: 1) displayed a warning icon in 2017 at the time of data collection, or 2) were not on the menu in 2017, but

were ordered in 2015 and contained >= 2,300 mg sodium.

**For items not on the menu in 2017, the 2015 sodium content is listed. Otherwise, the listed sodium content values are from 2017.

The following footnotes apply to items which list a range of sodium:

^a^ Jack Daniel's Grill/From the Grill - Pick 2 sides: 2 lowest sodium sides were Tomato Mozzarella Salad (220 mg) and Sweet Potato Fries (230 mg); 2 highest sodium sides were Fresh Spinach (570 mg) and Seasoned Fried (1,010 mg).

^b^ Wings: Lowest sodium flavor option was Garlic Parmesan; highest sodium flavor option was Sriracha w/Sriracha Ranch.

^c^ Sandwiches: Lowest sodium side was Side Salad with Low Fat Balsamic Vinaigrette (2017: 235 mg; 2015: 345 mg); highest sodium side was Seasoned Fries (2017: 1,010 mg; 2015: 980 mg).

^d^ Friday's Pick 2 Combo: 2 lowest sodium options were House Salad (280 mg) and Caesar Salad (590 mg); 2 highest sodium options were French Onion Soup (2,550 mg) and 1/2 California Chicken Club (1,830 mg).

^e^ Sliders - Choice of 2, 3 or 4: lowest sodium option includes 2 sliders; highest sodium option includes 4 sliders.

^f^ Kids' Menu - includes kids' beverage and 1 side: lowest sodium beverage was Lemonade (5 mg); highest sodium beverage was Chocolate Milk (135 mg). Lowest sodium side was Fruit Cup (0 mg); highest sodium side was Seasoned Fries (1,010 mg).

^g^ Your Call Burger: 1 cheese, 1 spread, 2 toppings: Lowest sodium cheese contained 120 mg sodium; highest sodium cheese contained 640 mg. Lowest sodium spread contained 60 mg sodium; highest sodium spread contained 85 mg. Lowest sodium topping contained 0 mg sodium; highest sodium topping contained 320 mg. Nutrition information provided by resturant only specified these ranges and therefore could not select 2 distinct lowest and highest sodium toppings; mutliplied each by 2 to apply to lowest and highest sodium ranges for this item.

ǂ although this item had < 2,300 mg sodium, it was labeled with a sodium warning icon at the time of data collection in 2017.

**S3 Table: High-sodium items*, Popeyes, 2015 and 2017**

| **Included Item*** | **Sodium content, mg**** | **Notes** |
| --- | --- | --- |
| 2 Piece Chicken Combo ^a, b^ | 1,480 - 3,965 |  |
| 3 Piece Chicken Combo ^a, b^ | 1,840 - 5,295 |  |
| 3 Piece Tender Combo ^a, c^ | 2,000 - 2,655 |  |
| 4 Piece Chicken Combo ^a, b^ | 2,200 - 6,625 |  |
| 5 Piece Chicken Combo ^a, d^ | 2,560 - 6,625 | only offered in 2015 |
| 5 Piece Tender Combo ^a, c^ | 2,827 - 3,555 |  |
| 5 Wings Combo ^a, e^ | 2,810 - 4,355 |  |
| 6 Biscuits | 2,700 |  |
| 8 Piece Chicken Family Meal ^f^ | 5,580 - 13,370 |  |
| 10 Piece Chicken ^d^ | 3,600 - 13,300 | only offered in 2015 |
| 11 Piece Chicken ^d^ | 3,960 - 14,630 | only offered in 2015 |
| 12 Biscuits | 5,400 |  |
| 12 Piece Chicken Family Meal ^g^ | 8,820 - 22,200 |  |
| 16 Piece Chicken Family Meal ^h^ | 12,060 - 30,190 |  |
| Cajun Fish Combo ^a^ | 1,310 - 1,855 |  |
| Chicken Tender Sandwich Combo ^i^ | 2,430 - 2,975 |  |
| Fish and Popcorn Shrimp Combo ^a^ | 3,190 - 3,735 |  |
| Popcorn Shrimp Combo ^a^ | 2,050 - 2,595 |  |

*included items that either: 1) displayed a warning icon in 2017 at the time of data collection, or 2) were not on the menu in 2017, but were ordered in 2015 and contained >= 2,300 mg sodium.

**For items not on the menu in 2017, the 2015 sodium content is listed. Otherwise, the listed sodium content values are from 2017.

^a^ For Tender, Fish, and Chicken Combos, lowest sodium version includes lowest sodium regular side (cole slaw), lowest sodium small drink (unsweetened or sweet tea), and 1 biscuit. Highest sodium version includes highest sodium regular side (mashed potatoes), highest sodium drink (Hawaiian Punch), and 1 biscuit.

^b^ Due to differences in data collection between 2015 and 2017, it was not possible to differentiate 2, 3 and 4 piece chicken combos in 2017. Thus, these are listed distinctly for 2015 and in a single category for 2017. The lowest sodium chicken piece (Spicy Leg, 360 mg) was multiplied by the # of pieces of chicken in the combo to the low sodium range for that combo; the same was done with the highest sodium chicken piece (Mild Breast, 1,330 mg) for the high sodium range for each combo.

^c^ Lowest sodium tenders (3 Spicy Tenders, 1240 mg/5 Spicy Tenders, 2067 mg) were used to calculate low sodium range. Highest sodium tenders (3 Mild Tenders, 1350 mg/5 Mild Tenders, 2,250 mg) were used to calculate high sodium range.

^d^ The lowest sodium chicken piece (Spicy Leg, 360 mg) was multiplied by the # of pieces of chicken in the combo to the low sodium range for that combo; the same was done with the highest sodium chicken piece (Mild Breast, 1,330 mg) for the high sodium range for each combo.

^e^ Lowest sodium wing (Spicy, 360 mg) was used to calculate low sodium range. Highest sodium wing (Mild, 610 mg) were used to calculate high sodium range.

^f^ 8 Piece Chicken Family Meal lowest sodium version includes lowest sodium large side (cole slaw) and 4 biscuits. Highest sodium option includes highest sodium large side (mashed potatoes) and 4 biscuits.

^g^ 12 Piece Chicken Family Meal lowest sodium version includes 2 lowest sodium large sides and 6 biscuits. Highest sodium option includes 2 highest sodium large sides and 6 biscuits.

^h^ 16 Piece Chicken Family Meal lowest sodium version includes 3 lowest sodium large sides and 8 biscuits. Highest sodium option includes 3. highest sodium large sides and 8 biscuits.

^i^ For Sandwich Combos, lowest sodium version includes lowest sodium regular side (cole slaw) and lowest sodium small drink (unsweetened or sweet tea). Highest sodium version includes highest sodium regular side (mashed potatoes) and highest sodium drink (Hawaiian Punch).

**S4 Table: High-sodium items*, Subway, 2015 and 2017**

| **Included Item*** | **Sodium content, mg**** |  |
| --- | --- | --- |
| B.L.T. Footlong | 2,260 |  |
| Bacon, Egg & Cheese Footlong | 2,610 |  |
| Big Hot Pastrami Melt Footlong | 2,940 |  |
| Black Forest Ham, Egg & Cheese Footlong | 2,280 |  |
| Buffalo Chicken Footlong | 2,200 |  |
| Chicken & Bacon Ranch Melt Footlong | 2,580 |  |
| Italian B.M.T. Footlong | 2,530 |  |
| Philly Cheesesteak Footlong | 2,560 | only offered in 2015 |
| Spicy Italian Footlong | 2,970 |  |
| Steak & Cheese Footlong | 2,060 |  |
| Steak, Egg & Cheese Footlong | 2,420 |  |
| Turkey Italiano Footlong | 2,980 | only offered in 2015 |
| Any other footlong sandwich purchased with "meal deal" (sandwich + 21 oz drink + Apples or 2 Cookies or Chips) ^a^ | 560 - 2,430 |  |

*included items that either: 1) displayed a warning icon in 2017 at the time of data collection, or 2) were not on the menu in 2017, but were ordered in 2015 and contained >= 2,300 mg sodium.

**For items not on the menu in 2017, the 2015 sodium content is listed. Otherwise, the listed sodium content values are from 2017.

^a^ Lowest sodium Meal Deal side combination included Apple Slices (0 mg) and Unsweetened Tea (0 mg). Highest sodium Meal Deal side combination included Nacho Doritos (360 mg) and Raspberry Tea (60 mg). Lowest sodium footlong sandwich was Veggie Delite (560 mg); highest sodium footlong sandwich that didn't display a sodium warning icon for the sandwich alone was the Cold Cut Combo (2,060 mg). Not all footlong sandwiches exceeded 2,300 mg sodium in combination with the highest sodium side and beverage, but at the time of data collection, the sodium warning icon, with a "Footlong only" clarification, was placed next to "Meal Deal" on the menu board, which applies to all sandwiches.

**S5 Table: Square root-transformed sodium and calorie content of dinnertime purchases at full-service and lunchtime purchases at quick-service restaurant chains, pre- (Baseline) and post- (Follow-up) implementation of the sodium warning icon in NYC**

|  | **NYC vs Yonkers** | | **NYC (intervention)** | | | | | | | **Yonkers (control)** | | | | | | |
| --- | --- | --- | --- | --- | --- | --- | --- | --- | --- | --- | --- | --- | --- | --- | --- | --- |
|  | **Difference In Difference** | **p value** | **Baseline** | | | **Follow-up** | | | **p value** | **Baseline** | | | **Follow-up** | | | **p value** |
| **Full-Service Restaurants** |  |  | n=734 | | | n=915 | | |  | n=418 | | | n=538 | | |  |
| Square root sodium, mean (95% CI) | -4.75 | **0.012** | 54.5 | (52.6, | 56.4) | 46.2 | (44.3, | 48.0) | **<.001** | 49.4 | (47.1, | 51.7) | 45.8 | (43.5, | 48.1) | **0.016** |
| Non-transformed sodium, mean (95% CI) | -524 | **0.011** | 3245 | (3036, | 3455) | 2279 | (2081, | 2477) | **<.001** | 2696 | (2452, | 2941) | 2254 | (2007, | 2500) | **0.008** |
| Square root calories, mean (95% CI) | -2.69 | **0.020** | 37.4 | (36.2, | 38.7) | 31.9 | (30.7, | 33.0) | **<.001** | 34.1 | (32.7, | 35.6) | 31.2 | (29.8, | 32.7) | **0.004** |
| Non-transformed calories, mean (95% CI) | -218 | **0.017** | 1524 | (1429, | 1618) | 1055 | (965, | 1146) | **<.001** | 1259 | (1149, | 1370) | 1009 | (898, | 1121) | **0.002** |
| **Quick-Service Restaurants** |  |  | n=338 | | | n=376 | | |  | n=317 | | | n=329 | | |  |
| Square root sodium, mean (95% CI) | 1.04 | 0.570 | 42.5 | (39.8, | 45.2) | 40.7 | (37.9, | 43.4) | 0.162 | 43.0 | (40.0, | 46.0) | 40.1 | (37.2, | 43.1) | 0.064 |
| Non-transformed sodium, mean (95% CI) | 258 | 0.185 | 1977 | (1709, | 2244) | 1777 | (1503, | 2051) | 0.131 | 2193 | (1834, | 2492) | 1735 | (1439, | 2031) | **0.010** |
| Square root calories, mean (95% CI) | -0.33 | 0.768 | 29.7 | (28.1, | 31.4) | 27.4 | (25.7, | 29.1) | **0.013** | 30.1 | (28.2, | 31.9) | 28.0 | (26.2, | 29.9) | **0.039** |
| Non-transformed calories, mean (95% CI) | 59 | 0.446 | 946 | (832, | 1059) | 794 | (677, | 911) | **0.018** | 1047 | (920, | 1174) | 836 | (710, | 962) | **0.006** |

Baseline data were collected between October 2015-January 2016. Follow-up data were collected between April-June 2017.

Models were adjusted for restaurant chain, gender, age group, education, and race/ethnicity, with random effects for restaurant location (street address).

Sample sizes for participants included in the models are shown. Due to missingness in fixed effects variables, 19 FSR and 8 QSR participants in the final analytic sample were not included in modeled estimates.

**Bolded** p values indicate statistically significant differences (p<0.05).

**S6 Table: High-sodium dinnertime purchases at IHOP and TGI Friday's and lunchtime purchases at Popeyes, pre- (baseline) and post- (follow-up) implementation of the sodium warning icon in NYC**

|  | **NYC vs Yonkers** | | **NYC (intervention)** | | | | | | | **Yonkers (control)** | | | | | | |  |  |
| --- | --- | --- | --- | --- | --- | --- | --- | --- | --- | --- | --- | --- | --- | --- | --- | --- | --- | --- |
|  | **Difference In Difference** | **p** | **Baseline** | | | **Follow-up** | | | **p** | **Baseline** | | | **Follow-up** | | | **p** |  |  |
| **IHOP** |  |  | N=627 | | | N=628 | | |  | N=146 | | | N=489 | | |  |  |  |
| # High sodium items purchased per participant, mean (95% CI) | -0.07 | 0.393 | 0.63 | (0.55, | 0.73) | 0.51 | (0.43, | 0.59) | **0.008** | 0.52 | (0.41, | 0.66) | 0.47 | (0.39, | 0.56) | 0.349 |  |  |
| Participants purchasing at least 1 high-sodium item, % (95% CI) | -0.3 | 0.964 | 52.0 | (44.8, | 59.2) | 49.2 | (41.5, | 56.9) | 0.412 | 47.5 | (35.8, | 59.5) | 45.0 | (36.8, | 53.4) | 0.652 |  |  |
| Participants purchasing at least 2 high-sodium items, % (95% CI) | -5.1 | 0.607 | 9.5 | (5.4, | 15.9) | 1.1 | (0.4, | 2.9) | **<.001** | 4.0 | (1.5, | 10.7) | 0.7 | (0.2, | 2.6) | **0.027** |  |  |
| **TGI Friday’s** |  |  | n=107 | | | n=287 | | |  | N=272 | | | N=49 | | |  |  |  |
| # High sodium items purchased per participant, mean (95% CI) | -0.26 | 0.212 | 1.09 | (0.80, | 1.48) | 0.85 | (0.65, | 1.12) | 0.085 | 0.89 | (0.69, | 1.16) | 0.91 | (0.58, | 1.43) | 0.900 |  |  |
| Participants purchasing at least 1 high-sodium item, % (95% CI) | -12.8 | 0.225 | 83.8 | (61.9, | 94.2) | 83.4 | (65.0, | 93.2) | 0.944 | 77.1 | (57.2, | 89.4) | 89.5 | (58.2, | 98.1) | 0.179 |  |  |
| Participants purchasing at least 2 high-sodium items, % (95% CI) | -7.0 | 0.624 | 18.2 | (5.1, | 47.9) | 6.1 | (1.5, | 22.0) | 0.053 | 10.0 | (2.8, | 29.9) | 4.9 | (0.4, | 40.7) | 0.385 |  |  |
| **Popeyes** |  |  | n=106 | | | n=145 | | |  | N=307 | | | N=168 | | |  |  |  |
| # High sodium items purchased per participant, mean (95% CI) | -0.1 | 0.524 | 0.45 | (0.25, | 0.84) | 0.48 | (0.27, | 0.88) | 0.587 | 0.48 | (0.27, | 0.82) | 0.56 | (0.31, | 1.01) | 0.155 |  |  |
| Participants purchasing at least 1 high-sodium item, % (95% CI) | -13.5 | 0.254 | 48.0 | (18.9, | 78.5) | 51.7 | (21.7, | 80.5) | 0.647 | 48.3 | (21.9, | 75.6) | 65.5 | (31.6, | 88.6) | 0.090 |  |  |

**Please note that the study was not sampled to assess differences by chain and interpret these results with caution.**

Baseline data were collected between October 2015-January 2016. Follow-up data were collected between April-June 2017.

Models were adjusted for gender, age group, education, and race/ethnicity, with random effects for restaurant location (street address).

Sample sizes for participants included in the models are shown. Due to missingness in fixed effects variables, some participants in the final analytic sample were not included in modeled estimates.

**Bolded** p values indicate statistically significant differences between baseline and follow-up.

Due to Subway’s small baseline sample size in Yonkers (N=10), results for Subway have not been included.

**S7 Table: Sodium content of dinnertime purchases at IHOP and TGI Friday's and lunchtime purchases at Popeyes, pre- (baseline) and post- (follow-up) implementation of the sodium warning icon in NYC**

|  | **NYC vs Yonkers** | | **NYC (intervention)** | | | | | | | **Yonkers (control)** | | | | | | |
| --- | --- | --- | --- | --- | --- | --- | --- | --- | --- | --- | --- | --- | --- | --- | --- | --- |
|  | **Difference In Difference** | **p** | **Baseline** | | | **Follow-up** | | | **p** | **Baseline** | | | **Follow-up** | | | **p** |
| **IHOP** |  |  | N=627 | | | N=628 | | |  | N=146 | | | N=489 | | |  |
| Square root sodium, mean (95% CI) | -3.13 | 0.118 | 52.0 | (50.0, | 53.9) | 43.0 | (40.9, | 45.2) | **<.001** | 48.8 | (45.5, | 52.2) | 43.1 | (40.8, | 45.4) | **0.006** |
| Non-transformed sodium, mean (95% CI) | -383 | 0.077 | 2964 | (2757, | 3172) | 1944 | (1724, | 2165) | **<.001** | 2603 | (2246, | 2960) | 1966 | (1726, | 2206) | **0.005** |
| **TGI Friday’s** |  |  | N=107 | | | N=287 | | |  | N=272 | | | N=49 | | |  |
| Square root sodium, mean (95% CI) | -5.55 | 0.297 | 57.5 | (49.6, | 65.4) | 51.3 | (45.2, | 57.5) | 0.099 | 52.4 | (46.5, | 58.4) | 51.8 | (40.5, | 63.1) | 0.876 |
| Non-transformed sodium, mean (95% CI) | -523 | 0.383 | 3585 | (2689, | 4482) | 2832 | (2152, | 3512) | 0.088 | 3040 | (2376, | 3705) | 2810 | (1509, | 4111) | 0.619 |
| **Popeyes** |  |  | N=106 | | | N=145 | | |  | N=307 | | | N=168 | | |  |
| Square root sodium, mean (95% CI) | 1.05 | 0.755 | 46.8 | (36.0, | 57.6) | 45.1 | (34.8, | 55.4) | 0.545 | 47.8 | (38.8, | 56.8) | 45.1 | (34.7, | 55.4) | 0.264 |
| Non-transformed sodium, mean (95% CI) | 247 | 0.517 | 2414 | (1252, | 3575) | 2162 | (1052, | 3272) | 0.427 | 2614 | (1642, | 3586) | 2115 | (995, | 3236) | 0.123 |

**Please note that the study was not sampled to assess differences by chain and interpret these results with caution.**

Baseline data were collected between October 2015-January 2016. Follow-up data were collected between April-June 2017.

Models were adjusted for gender, age group, education, and race/ethnicity, with random effects for restaurant location (street address).

Sample sizes for participants included in the models are shown. Due to missingness in fixed effects variables, some participants in the final analytic sample were not included in modeled estimates.

**Bolded** p values indicate statistically significant differences between baseline and follow-up.

Due to Subway’s small baseline sample size in Yonkers (N=10), results for Subway have not been included.
